# Effect of continual quality improvement of palliative care consultation teams by iterative, customer satisfaction survey-driven evaluation

**DOI:** 10.1186/s12904-021-00741-2

**Published:** 2021-03-19

**Authors:** Noriyuki Kawabata, Mikio Nin

**Affiliations:** Department of Palliative Care, Osaka Rosai Hospital, 1179-3 Nagasone-cho, Kita-ku, Sakai City, Osaka, Japan

**Keywords:** Palliative care consultation team, Quality improvement, Periodic evaluation, Customer satisfaction

## Abstract

**Background:**

Current consensus recognizes the benefits of early intervention in palliative care consultation teams (PCCTs). As consultants, we should now attempt to improve the quality of our teams by utilizing a method mainly used in the business field. We aimed to investigate the effects of iterative evaluation of customer satisfaction surveys, filled by physicians and ward nurses in this study, for quality improvement of PCCTs.

**Methods:**

In October 2019, the participants filled the first questionnaire survey about palliative care and PCCTs at a 678-bed hospital, and improvement areas were uncovered. Refinements were planned and implemented, and then reevaluated using the second questionnaire survey in March 2020.

**Results:**

In addition to the characteristics of our clients evaluated from approximately 500 valid responses, the first survey showed that the response rate of the questionnaire, knowledge of palliative care and PCCTs, and publicity of the PCCT were recognized as issues needing attention. We planned to contrive ways to collect questionnaires, hold monthly workshops for palliative care, launch newsletters of palliative care, and go on client rounds. The second survey revealed improvements in the physicians’ response rate (*p* = 0.02), the accuracy rate of application of PCCTs in Japan (*p* < 0.01), and ward nurses’ confidence in opioid use (*p* = 0.04) and tendency toward easier accessibility to the PCCT (*p* = 0.07).

**Conclusion:**

Continual quality improvements through iterative, customer satisfaction survey-driven evaluation are a widely established practice in the business field. By using this appropriately, we could enable PCCTs to improve their quality.

**Supplementary Information:**

The online version contains supplementary material available at 10.1186/s12904-021-00741-2.

## Background

With the rapidly aging world population, we will require much more palliative care in future [1]. The appropriate introduction and modality of palliative care have been researched [2–4]. Palliative care consultation teams (PCCTs) play a large part in palliative care settings, conventionally comprising multidisciplinary members, such as physicians, nurses, pharmacists, and so forth. Based on requests from clients, who are usually attending physicians and ward nurses, PCCTs provide assistance for symptom management, establishing care goals, treatment decisions, advanced care planning, and other issues associated with living with serious illness [5]. PCCTs have been increasingly available in many countries, and their impact has been evaluated since 2000 [6–8]. According to these reports, PCCTs have proved their conduciveness for inpatients and outpatients in diverse cultural backgrounds [9–11]. Despite the diverse results reported, current guidelines of the American Society of Clinical Oncology and the European Society for Medical Oncology recommend the early introduction of palliative care and PCCTs [12, 13]. Lately, there have been reports regarding quality improvement of PCCTs by using self-check questionnaires [14], and repeated aggregation of expertise, that is, the Delphi method [15].

In terms of quality improvement, quality control of processes, products, and services is mandatory in the business field, and some methods introduced as frameworks are Plan-Do-Check-Act (PDCA) cycle and its derivatives [16], Observe-Orient-Decide-Act (OODA) loop [17], and Kaizen [18]. Utilizing of these frameworks has also been explored and evaluated in other healthcare fields [19, 20]. Although these frameworks differ in size, speediness of decision-making, and intervals of reevaluations, their core components share critical and result-driven planning. There is a need for critical assessment under varied situations and identification of the gaps between ideal and real conditions, planning for better outcomes, and performing refinements based on the plan. Moreover, from the perspective of a consultancy, we believe that team evaluation should contain appraisals not from patients and caregivers as end users, but clients, as in customer satisfaction surveys. To our knowledge, there is no previous research on quality improvements of PCCTs including iterated evaluations from clients. Therefore, for universal interests and translation of the business technique to improve PCCTs’ quality, the primary objective of the present study was to examine whether periodic assessments of PCCTs from attending physicians and ward nurses, make the teams more effective.

## Methods

### Ethical considerations

The institutional review board of Osaka Rosai Hospital approved the study protocol (receipt number 31–53, September 13, 2019, and receipt number 31–109, February 20, 2020). The participants were assured that the data would be anonymous, and that they could, without prejudice, withdraw from the study at any time.

### Participants

Osaka Rosai Hospital is a 678-bed hospital in the Osaka prefecture of Japan. In Japan, the usual clients of PCCTs are physicians and nurses. Physicians from all departments with a history of PCCT requests, and nurses from all wards were enrolled. Doctors from the departments of radiology, orthopedics, ophthalmology, dermatology, pediatrics, and rehabilitation were excluded. A total of 127 physicians and 423 ward nurses participated in the first step of the study.

### Study design

We planned two-step quantitative research, in a single institute, using questionnaires to determine the effectiveness of quality improvements of a PCCT that reorganized in April 2019. The first survey uncovered the characteristics of the clients of the PCCT and the issues needing improvements. Next, new strategies and tactics were developed to resolve the highlighted problems. We conducted a second survey and compared its results with those of the first. The first step of the research was conducted over 2 weeks starting from October 1, 2019. The second step, using the second questionnaire, originally planned for 6 months later, was modified to be administered 5 months later because personnel transfer takes place in the latter part of March in Japan. Therefore, conduction started from March 2, 2020, and continued for 2 weeks.

### Questionnaires

We developed a questionnaire for the first survey (Appendix 1). Questions (Qs) 1 and 2 requested information on respondents’ characteristics. Q3 and Q4 concerned their knowledge of permitted indication of patients and diseases for PCCTs in Japan. Q5 and Q6 assessed the respondents’ general recognition of palliative care. Questions 7 to 9 assessed the respondents’ self-confidence for management in challenging situations in palliative care as healthcare providers. Q10 and Q11 asked how the clients behave in clinical settings. Questions 5 to 11 were aimed to unveil the real situation of our clients. Questions 12 to 15, and Q17 were related to the appraisal of the PCCT and the relationship between the PCCT and the clients. Q16 assessed the respondents’ interests and needs. Questions 12 through 17 were set to discover the strengths and weaknesses of our team for improvement strategies. Q18 and Q19 were related to issues likely to arise because only physicians can prescribe medicine in Japan, and conflicts that may occur when the opinions of the attending physician and others clash as contextual elements; regarding our consultation system in Japan, we work for patients under requests by clients. Q20 assessed whether there is a demand for educational opportunities to avoid self-absorption workshops.

We made minor changes to the questionnaire to better understand the results of quality improvement derived from the first survey. Q16 was deleted, and Q20 and Q21 were related to the clients’ attendance at workshops and the reasons for not attending (Appendix 2).

Altogether, these questions were reflected to answer central components as in SQUIRE 2.0, a guideline for quality improvement: why we started the study, what we did, what we found, and what it means [21].

### Statistical analysis

The primary outcome was the impact of strategies and tactics to improve the PCCT. Reported changes in the questionnaires were analyzed using Welch’s t-test because the two questionnaires had a different number of respondents. To understand the nature of the clients, the correlation coefficients were determined by polychoric correlations in Likert scales, which are normally considered ordinal and discrete scales [22]. Here, strength of correlation (rho) was based on the following criteria: 1.0 to 0.7 = strong, 0.7 to 0.4 = moderate, and 0.4 to 0.1 = weak [23]. Comparison of binary data used a Chi-square test, while Fisher’s exact test was applied in the case of expected values of five or lower. Statistical significance was set at < 0.05. Analyses were performed using R version 3.5.2 (R Core Team, Vienna, Austria).

## Results

Of the 127 physicians and 423 ward nurses, 101 physicians (79.5%) and 394 nurses (93.1%) gave valid responses in the first survey (Table 1). To select the questions that we regarded as related to each other, combinations of questions revealed the rho was 0.4 or more (Table 2). Regarding the questions related to practice (Q7, Q8, and Q9), both physicians and nurses showed relatively strong correlations (rho = 0.55 to 0.80). The results of the first survey revealed a part of our clients’ beliefs and experiences with PCCTs: the physicians who recognized the PCCT as a useful consultant had experienced troubles in symptom management, felt free to request the PCCT, and had required workshops about palliative care; the ward nurses who had easily accessed the PCCT had found the team helpful. Those who wanted to take part in palliative care workshops tended to have problems with symptom control and evaluated themselves as good listeners to their patients. Self-evaluation of confidence for maneuvering opioids, hydration in the end stage of life, and sedation based on references were strongly and positively correlated with each other in both physicians and ward nurses.

**Table 1:** Demographics of participants in the first survey

	Physicians				Ward nurses			
Response rate	101/127 (79.5%)				394/423 (93.1%)			
Years of professional experience	1 ~ 4	5 ~ 9	10 ~ 14	15~	1 ~ 4	5 ~ 9	10 ~ 14	15~
	25.7%	20.8%	19.8%	33.7%	30.3%	18.3%	12.5%	38.9%

**Table 2:** Combinations of questions in the first survey with moderate correlations

Physicians	rho
Q10 & Q17	0.59
Q15 & Q17	0.49
Q17 & Q20	0.45
**Ward nurses**	**rho**
Q10 & Q20	0.53
Q11 & Q20	−0.42
Q15 & Q17	0.41
Q15 & Q20	0.42
Q18 & Q19	0.46

Rho: the correlation coefficient by polychoric correlation

From the first survey, the following three aspects of the PCCT needed improvements.

1. The response rates of the questionnaire from major clients, that is, gastroenterology (61.5%) and cardiology (66.7%), a ward mainly involved in abdominal surgery (62.2%), and interns (45.8%) with less than 2 years of experience as physicians, our targets, were relatively low, although the overall response rates were high (79.5% in the physicians, and 93.1% in the ward nurses).
2. Knowledge of palliative care and PCCTs including target diseases and recommended treatment described in authorities e.g., guidelines and textbooks was inadequate regardless of job category and years of professional experience, although the clients expressed a need for palliative care in our hospital (76.3%). The accuracy rate of permitted application for PCCTs regulated by the government was 44.8%. In addition, 46.0% of the physicians and 33.3% of the ward nurses felt unconfident regarding palliative care; this did not correlate with the length of professional experience (rho (physicians) = 0.06; rho (ward nurses) = 0.28). Overall rates of confidence in prescription and management of opioids, hydration at the end stage, and sedation based on authorities revealed unexpectedly low percentages (50.6, 32.0, and 29.7%, respectively).
3. Publicity of our PCCT and palliative care for client candidates were still insufficient (changes in the PCCT activity for the latest 6 months noted at 56.2%), although user satisfaction was notably high (91.7%). Moreover, some clients had faced difficulty in requesting the PCCT (11.3%, 9 physicians and 33 ward nurses).

We formulated the following strategies to address these issues.

1. We designed ways to collect questionnaires. When our team assistants distributed the questionnaires, they attended a physician’s meeting and collected the filled questionnaires on site. Additionally, an announcement about the survey was made at nurse meetings held every morning.
2. Workshops were held each month about opioids, dyspnea, anxiety/insomnia, and general fatigue, which were the top five symptoms identified through Q16, as well as sedation, in which the healthcare providers had the lowest confidence (Q7 to Q9).
3. Bimonthly internal newsletters on palliative care and the PCCT activity were launched. Furthermore, we made daily rounds to our customers to obtain opportunities for in-person communication and acquaintance with our members in all the wards.

All the aforementioned strategies and tactics were implemented except the workshops about sedation and general fatigue, which were canceled due to the COVID-19 spread. Remaining workshops were conducted by physicians and a psychiatrist of PCCT members.

The results of the improvements are as follows (Table 3):

1. The response rate improved from 79.5 to 90.6% (*p =* 0.02) in physicians and from 93.1 to 95.7% (*p =* 0.15) in ward nurses. Examining the breakdown, we found that the response rates of the abdominal surgery ward (88.9%, *p =* 0.01, 95% confidence interval (CI): 0.04 to 0.78), and interns (87.5%, *p* < 0.01, 95% CI: 0.02 to 0.59) significantly improved. Gastroenterology (69.2%, *p =* 1.00, 95% CI: 0.10 to 4.75) and cardiology (77.8%, *p =* 0.71, 95% CI: 0.10 to 3.14) exhibited no changes.
2. After the workshops, the accuracy rate of permitted applications for PCCTs rose to 66.7% (*p* < 0.01). Self-evaluation of the proper use of opioids indicated a small improvement in ward nurses (t(786.9) = 2.1, *p =* 0.04, 95% CI: 0.01 to 0.21, mean ± standard deviation: 2.52 ± 0.75 to 2.41 ± 0.73), but not in the physicians (t(201.7) = − 0.1, *p* = 0.92, 95% CI: − 0.26 to 0.24).
3. A notice regarding changes in PCCT activities in Q12 found no significant changes (*p =* 0.48). Easy accessibility to the PCCT indicated no significant changes in the physicians (t (132.1) = − 0.37, *p =* 0.71, 95% CI: − 0.42 to 0.29); however, ward nurses (t (655.6) = 1.8, *p =* 0.07, 95% CI: − 0.01 to 0.24) showed an improvement (2.37 ± 0.82 to 2.25 ± 0.78).

**Table 3:** Highlighted differences of the second survey from the first survey

	Physicians	Ward nurses
Response rate	79.5 to 93.1% (*p* = 0.02)	93.1 to 95.7% (*p* = 0.15)
Accuracy rate of PCCTs permitted applications	47.0 to 75.2% (*p* < 0.01)	44.3 to 64.0% (*p* < 0.01)
Self-evaluation of proper use of opioids	*p* = 0.92	*p* = 0.04
Easy accessibility to the PCCT	*p* = 0.71	*p* = 0.07

Only a proportion of those who expressed a desire to attend workshops participated in them—20 physicians (17.2%) and 131 ward nurses (32.9%)––mainly because of the lack of a convenient time to attend (66.7% in the physicians and 65.7% in the ward nurses). Moreover, we discerned perception gaps between the physicians and the ward nurses in troubles related to the PCCT (Q17 and Q18) in both surveys (*p* < 0.01). The ward nurses not only perceived different opinions between the attending physicians and the PCCT, but also saw the time lag for patient application as more problematic than the physicians. No other questions showed significant changes.

## Discussion

We found statistical improvements over 5 months in accessing the PCCT application regulated by the government, and familiarity with opioid use according to ward nurses’ authorities. In addition, ward nurses indicated signs of easier access to the PCCT. No change in other answers to the second questionnaire worked as noninterventional control.

Newly emerging and intractable issues were identified, and suitable solutions were implemented. For example, an overall improvement in the response rate to the second survey was achieved; however, responses from the department of gastroenterology and cardiology were poor, suggesting the impracticality of the method that we had used with the physicians for 3 or more years. We conjectured that they required a different impetus to respond because requests to the PCCT from the department of gastroenterology had been the second most common; there were few from the department of cardiology. Therefore, we tried to convey how important the responses to the questionnaires were to improve the PCCT activity in the department of gastroenterology, and how useful the PCCT was for consultation with the department of cardiology. We also recognized that only a small proportion of those who were interested in the workshops could participate in them, and thus, we held multiple workshops on each theme.

We understand that the exclusion of patients and caregivers from our subjects might feel strange; however, our intention was to emphasize the employer-employee relationship in consultations and a priority for patients appertains to clients [5] in our settings. For this reason, we experienced occasional conflicts with clients, and developed Q18 and Q19 to evaluate the clash. The present study did not aim to develop a standardized, validated tool for quality improvement for use in any situation. A limitation of the study was the reproducibility and reliability of the results obtained using our non-standard questionnaires. Apart from ours, there have been no previous studies to indicate that clients continuously evaluated PCCTs; this was expected because the questionnaires were constructed specifically for our PCCT. The most common guideline, SQUIRE 2.0 [21], and a review [24] to improve quality emphasize local context and the difficulty in generalizing results from such research. As cultural and legal differences may cause unanticipated obstacles when a strategy is simply translated to other settings [25], and frameworks have been noted to have some downsides—for instance, a planning paralysis can be problematic in the plan phase of the PDCA cycle [20]—we believe that this iterative quality control approach derived from customer survey is applicable to any organization and country. For instance, while a startup PCCT like ours might aim at publicity, a well-established PCCT might try to identify new needs and improve efficiency. Using this method, finely tuned interventions based on different settings and needs could deliver results. As another concern, satisfaction is derived from the relationship expectation of subjects and results, and therefore the evaluation does not contribute to the overall improvement of PCCTs. However, we believe that consultation teams should not ignore any clients’ satisfaction whatsoever. Furthermore, the methodology has been universally established in the business field. Although an insufficient solution could result in failure to improve the expected [24], in the current business setting involving uncertainty, we are aware that solutions to address problems uncovered by surveys cannot be perfect. Rather, by being too meticulous in considering solutions, we may encounter two common pitfalls: to consume tremendous amounts of time in the plan phase—the so-called planning paralysis—or not being able to implement the strategies due to overcomplicated plans.

Additionally, there are no perfect frameworks for quality improvement, and we have not referred to or recommended a specific framework such as the PDCA cycle or OODA loop so far. From the business perspective, effectual frameworks are said to vary in research sizes, require reevaluation cycles, and leadership that endorses and orients repeated surveys. Again, if specific strategies and tactics do not function as well as expected, we should determine the causes through a reevaluation phase and should not hesitate to change the previously employed strategy. At the same time, strategies and tactics should be critically evaluated, balanced by easy concessions and feasibility, for the improvements to be functional.

## Conclusions

In conclusion, the current consensus in palliative care settings recognizes the usefulness of PCCTs and we should now shift to investigating how to polish the quality of PCCTs. To this end, we employed a well-established strategy mainly used in the business field: repeated data-driven improvement and checks as consultants. This is a sustainable and useful method for continual quality improvement of PCCTs in various contexts.

## Supplementary Information

**Additional file 1.**

## Data Availability

The datasets generated and/or analyzed during the current study are not publicly available due to using paper-based questionnaires but are available from the corresponding author on reasonable request.
