# Supplementary material for: Effect of continual quality improvement of palliative care consultation teams by iterative, customer satisfaction survey-driven evaluation
Source: BMC Palliat Care. 2021 Mar 19;20:46. doi: 10.1186/s12904-021-00741-2 (PMC7978439; doi:10.1186/s12904-021-00741-2)
Supplement: Supplementary file 1 — Additional file 1. [file 12904_2021_741_MOESM1_ESM.docx]

Appendix. 1 The questionnaire used in the first survey

The palliative care consultation team (PCCT) is dedicated to customer satisfaction. Through this questionnaire, your responses will be helpful in enhancing the quality of our team and in understanding your recognition of palliative care. This is performed with the permission of the Institutional Review Board, and the results of the questionnaires will be potentially used in a future study without identifying personal information.

If this is not acceptable, please check the following sentence and return this questionnaire.

I do not answer because I do not want to answer for any reason.

Q1. Where are you currently working? Doctors indicate the department, and nurses indicate the ward.

Q2. How many years of experience do you have as a medical professional? Circle ONE option.

1. 1 to 4 years 2. 5 to 9 years 3. 10 to 14 years 4. 15 years or more

Q3. Do you know which diseases are eligible for consulting PCCT? Cancers or end-stage heart failure? Circle ONE option.

1. Both 2. Only cancer 3. Only end-stage heart failure 4. Neither

Q4. Do you know that early involvement of palliative care is recommended regardless of a do-not-resuscitate order or the best supportive care conditions? Circle ONE option.

1. Yes 2. No

Q5. Do you feel that an acute care hospital like ours does not need palliative care, including symptom management and terminal care? Circle ONE option.

1. Strongly agree 2. Agree 3. Neutral 4. Disagree 5. Strongly disagree

6. I am not sure because of the non-engaged diseases I deal with

Q6. Are you weak in palliative care? Circle ONE option.

1. Strongly agree 2. Agree 3. Neutral 4. Disagree 5. Strongly disagree

6. I am not sure because of the non-engaged diseases I deal with

Q7. Do you order or prescribe opioids based on textbooks or guidelines? Circle ONE option.

1. Strongly agree 2. Agree 3. Neutral 4. Disagree 5. Strongly disagree

Q8. Do you administer drips in the terminal phase based on textbooks or guidelines? Circle ONE option.

1. Strongly agree 2. Agree 3. Neutral 4. Disagree 5. Strongly disagree

Q9. Do you perform sedation based on textbooks or guidelines? Circle ONE option.

1. Strongly agree 2. Agree 3. Neutral 4. Disagree 5. Strongly disagree

Q10. Have you experienced trouble with symptom management? Circle ONE option.

1. Strongly agree 2. Agree 3. Neutral 4. Disagree 5. Strongly disagree

Q11. Are you too busy to listen heartily to the patient? Circle ONE option.

1. Strongly agree 2. Agree 3. Neutral 4. Disagree 5. Strongly disagree

Q12. Have you noticed changes in PCCT activity (members and frequency) since April? Circle ONE option.

1. Yes 2. No 3. I do not know because I have never been offered

Q13. Have you experienced changes in the proposals from PCCT for consultation since last April? Circle ONE option.

1. Strongly agree 2. Agree 3. Neutral 4. Disagree 5. Strongly disagree

6. I do not know because I have never been offered 7. I am not sure because I had enrolled before

Q14. Have you observed changes in your attitude toward palliative care by PCCT since April? Circle ONE option.

1. Strongly agree 2. Agree 3. Neutral 4. Disagree 5. Strongly disagree

6. I do not know because I have never been offered

Q15. Do you feel comfortable to consult the PCCT? Circle ONE option.

1. Strongly agree 2. Agree 3. Neutral 4. Disagree 5. Strongly disagree

6. I do not know because I have never been offered

Q16. What are the symptoms you need to consult? Circle ALL relevant options.

1. Pain 2. Dyspnea 3. Fatigue 4. Appetite loss 5. Depression 6. Insomnia

7. Anxiety 8. Delirium 9. I do not know because of the non-engaged diseases I deal with

10. Others ( )

Q17. Is the PCCT serviceable to you now? Circle ONE option.

1. Strongly agree 2. Agree 3. Neutral 4. Disagree 5. Strongly disagree

6. I do not know because I have never been offered

Q18. Have you experienced any trouble due to differences between your intention or that of the doctor in charge and a PCCT proposal? Circle ONE option.

1. Strongly agree 2. Agree 3. Neutral 4. Disagree 5. Strongly disagree

6. I do not know because I have never been offered

Q19. Have you experienced any trouble because there is a time lag between a PCCT proposal and action? Circle ONE option.

1. Strongly agree 2. Agree 3. Neutral 4. Disagree 5. Strongly disagree

6. I do not know because I have never been offered

Q20. Would you like to participate in a palliative care workshop if held? Circle ONE number.

1. Strongly agree 2. Agree 3. Neutral 4. Disagree 5. Strongly disagree

6. I do not know because of the non-engaged diseases I deal with

Please provide your comments and suggestions regarding PCCT.

Thank you for your cooperation.

Appendix. 2 The questionnaire used in the second survey

The palliative care consultation team (PCCT) is dedicated to customer satisfaction. Through this questionnaire, your responses will be helpful in enhancing the quality of our team and in understanding your recognition of palliative care. This is performed with the permission of the Institutional Review Board, and the results of the questionnaires will be potentially used in a future study without identifying personal information.

If this is not acceptable, please check the following sentence and return this questionnaire.

I do not answer because I do not want to answer for any reason.

Q1. Where are you currently working? Doctors indicate the department, and nurses indicate the ward.

Q2. How many years of experience do you have as a medical professional? Circle ONE option.

1. 1 to 4 years 2. 5 to 9 years 3. 10 to 14 years 4. 15 years or more

Q3. Do you know which diseases are eligible for consulting PCCT? Cancers or end-stage heart failure? Circle ONE option.

1. Both 2. Only cancer 3. Only end-stage heart failure 4. Neither

Q4. Do you know that early involvement of palliative care is recommended regardless of a do-not-resuscitate order or the best supportive care conditions? Circle ONE option.

1. Yes 2. No

Q5. Do you feel that an acute care hospital like ours does not need palliative care, including symptom management and terminal care? Circle ONE option.

1. Strongly agree 2. Agree 3. Neutral 4. Disagree 5. Strongly disagree

6. I am not sure because of the non-engaged diseases I deal with

Q6. Are you weak in palliative care? Circle ONE option.

1. Strongly agree 2. Agree 3. Neutral 4. Disagree 5. Strongly disagree

6. I am not sure because of the non-engaged diseases I deal with

Q7. Do you order or prescribe opioids based on textbooks or guidelines? Circle ONE option.

1. Strongly agree 2. Agree 3. Neutral 4. Disagree 5. Strongly disagree

Q8. Do you administer drips in the terminal phase based on textbooks or guidelines? Circle ONE option.

1. Strongly agree 2. Agree 3. Neutral 4. Disagree 5. Strongly disagree

Q9. Do you perform sedation based on textbooks or guidelines? Circle ONE option.

1. Strongly agree 2. Agree 3. Neutral 4. Disagree 5. Strongly disagree

Q10. Have you experienced trouble with symptom management? Circle ONE option.

1. Strongly agree 2. Agree 3. Neutral 4. Disagree 5. Strongly disagree

Q11. Are you too busy to listen heartily to the patient? Circle ONE option.

1. Strongly agree 2. Agree 3. Neutral 4. Disagree 5. Strongly disagree

Q12. Have you noticed changes in PCCT activity (members and frequency) since April? Circle ONE option.

1. Yes 2. No 3. I do not know because I have never been offered

Q13. Have you experienced changes in the proposals from PCCT for consultation since last April? Circle ONE option.

1. Strongly agree 2. Agree 3. Neutral 4. Disagree 5. Strongly disagree

6. I do not know because I have never been offered 7. I am not sure because I had enrolled before

Q14. Have you observed changes in your attitude toward palliative care by PCCT since April? Circle ONE option.

1. Strongly agree 2. Agree 3. Neutral 4. Disagree 5. Strongly disagree

6. I do not know because I have never been offered

Q15. Do you feel comfortable to consult the PCCT? Circle ONE option.

1. Strongly agree 2. Agree 3. Neutral 4. Disagree 5. Strongly disagree

6. I do not know because I have never been offered

Q16. Is the PCCT serviceable to you now? Circle ONE option.

1. Strongly agree 2. Agree 3. Neutral 4. Disagree 5. Strongly disagree

6. I do not know because I have never been offered

Q17. Have you experienced any trouble due to differences between your intention or that of the doctor in charge and a PCCT proposal? Circle ONE option.

1. Strongly agree 2. Agree 3. Neutral 4. Disagree 5. Strongly disagree

6. I do not know because I have never been offered

Q18. Have you experienced any trouble because there is a time lag between a PCCT proposal and action? Circle ONE option.

1. Strongly agree 2. Agree 3. Neutral 4. Disagree 5. Strongly disagree

6. I do not know because I have never been offered

Q19. Would you like to participate in a palliative care workshop if held? Circle ONE number.

1. Strongly agree 2. Agree 3. Neutral 4. Disagree 5. Strongly disagree

6. I do not know because of the non-engaged diseases I deal with

Q20. We held monthly workshops about pain/opioids, dyspnea, anxiety/insomnia, sedation, and fatigue management; the last two topics were canceled due to COVID-19 spread. Circle ONE option.

1. I participated 2. I did not participate

Q21. If not, please circle the ONE option indicating the reason.

1. I did not intend to participate 2. I have no time 3. I did not notice the workshops

4. The topics were not relevant for me 5. Others ( )

Please provide your comments and suggestions regarding PCCT.

Thank you for your cooperation.
